# The Incidence of the Agenesis of Fetal Ductus Venosus at the 11–13 Weeks’ Ultrasound Examination

**DOI:** 10.7759/cureus.31748

**Published:** 2022-11-21

**Authors:** Athena Souka, Panagiotis Antsaklis, Konstantinos Tasias, Zacharias Fasoulakis, George Daskalakis

**Affiliations:** 1 1st Department of Obstetrics and Gynecology, National and Kapodistrian University of Athens School of Medicine, Athens, GRC; 2 1st Department of Obstetrics and Gynecology, National and Kapodistrian University of Athens, Athens, GRC

**Keywords:** ultrasound, noonan syndrome, first trimester, ductus venosus, agenesis

## Abstract

Objective: The objective is to examine the incidence of agenesis of fetal ductus venosus (DV) at the routine ultrasound examination at 11-13 weeks.

Materials and methods: This is a retrospective study on women presenting for screening for chromosomal abnormalities. The fetal DV was routinely examined by color Doppler in the sagittal view.

Results: Out of 8,304 fetuses examined, there were 13 cases of DV agenesis (0.15%). The umbilical vein drainage was intra-hepatic in two-thirds of the cases, and all resulted in normal live births. In the remaining one-third of cases, the umbilical vein drained to the inferior vena cava and all had a poor outcome because of aneuploidies, cardiac defects, and Noonan syndrome.

Conclusion: Fetal DV agenesis occurs in about one in 650 fetuses and the majority of cases have a benign course and a favorable outcome. Failure to identify the DV should prompt a detailed ultrasound examination, identification of the drainage site of the umbilical vein, and genetic testing.

## Introduction

The ductus venosus (DV) is a vascular shunt situated within the fetal liver, connecting the umbilical vein (UV) to the inferior vena cava, and plays a major role in the distribution of highly oxygenated umbilical venous blood to the heart. The DV is one of the fetal circulation shunts that constitute the adaptive mechanisms in conditions of fetal hypoxia and closes in the first two to 18 days after birth [1,2].

Until the beginning of the 2000s, and despite its importance, DV was not routinely studied in first-trimester ultrasound examinations, even in cases when there was severe growth restriction [3]. However, during the last few years, DV flow doppler examination has been added to the first-trimester ultrasound and takes part in risk assessment for fetal anomalies, since studies have proven that abnormal DV is associated with chromosomal abnormalities [4,5]. However, the majority of the literature consists of case reports or case series, and only a few studies report on the prevalence of DV agenesis in routine obstetric populations, mainly because first-trimester assessment of DV is technically difficult and time-consuming [5-11]. The present study aims to assess the prevalence of the condition in the general obstetric population and to report the outcomes of the pregnancies identified with DV agenesis.

## Materials and methods

The database of two fetal medicine units of the 1st Department of Obstetrics and Gynecology was searched for pregnancies presenting for routine first-trimester assessment at 11-13 weeks between 2009 and 2020. Cases referred for a second opinion because of ultrasound findings and/or abnormalities were excluded.

The screening protocol for the 11-13 weeks ultrasound scan included measurements of the fetal crown-rump length (CRL) and the nuchal translucency (NT), assessment of the presence of the nasal bone, assessment of the flow in the tricuspid valve of the fetal heart and the flow in the DV. Fetal anatomy was examined following the protocols of the International Society of Obstetric and Gynecological Ultrasound (ISUOG) and the Hellenic Society of Ultrasound. In addition, the color Doppler was used to visualize the chambers and the great vessels of the heart. The data were recorded in a computer database at the time of the examination (Astraia software).

**Figure 1:**
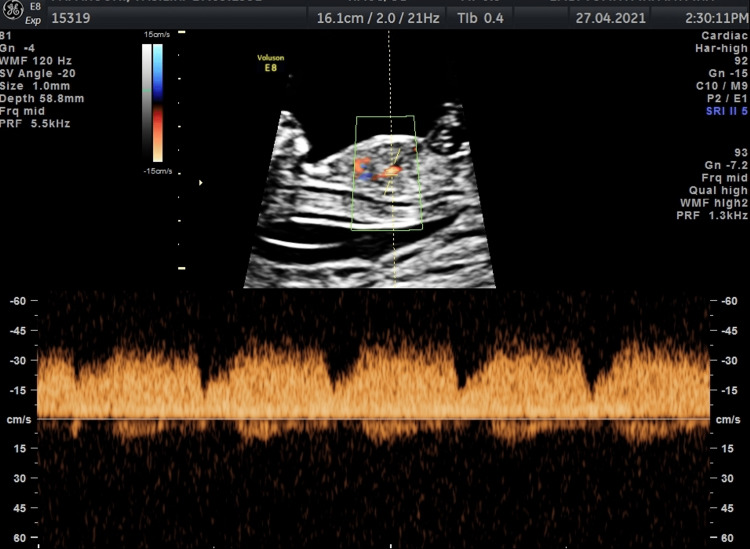
Fetal ductus venosus flow assessment. The ventral mid-sagittal view of the fetus is obtained and magnified so that the fetal thorax and abdomen occupy the screen.

The DV was assessed in the longitudinal view of the fetus and according to the guidelines proposed by the Fetal Medicine Foundation (FMF). Briefly, a ventral mid-sagittal view of the fetus is obtained and magnified so that the fetal thorax and abdomen occupy the screen (Figure 1). Color Doppler is applied and the UV, the DV, and the fetal heart are seen. The insonation angle should be less than 30◦ and the sample volume should be 0.5 to 1mm. In the first five years of the screening period, the DV was assessed only for the presence, absence, or reversal of the a-wave. After 2012 the pulsatility index of the DV waveform was measured and recorded.

## Results

**Figure 2:**
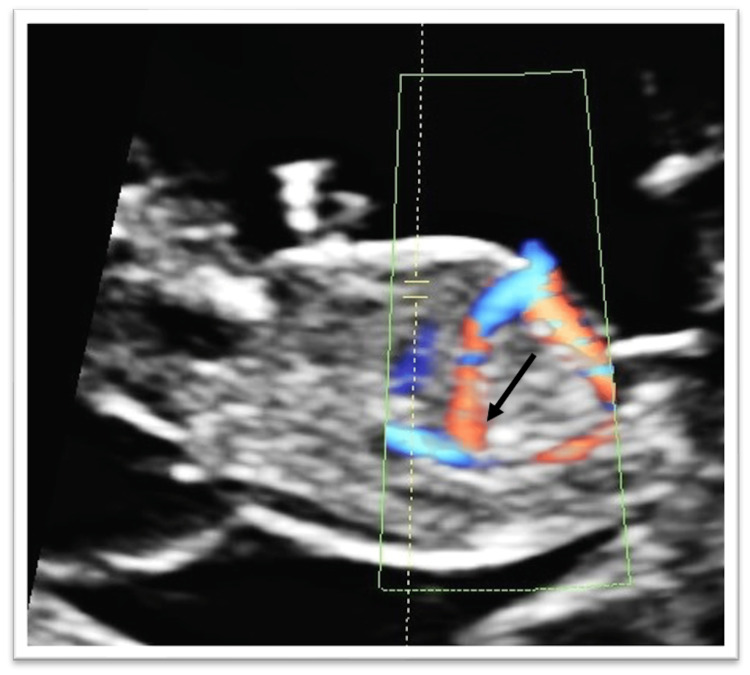
Agenesis of the ductus venosus: umbilical vein draining directly to the inferior vena cava (black arrow).

During the study period, 8,304 fetuses were screened and 13 had agenesis of the DV (0.15%). The cases with agenesis of the DV are presented in Table 1. In nine out of 13 DV agenesis cases (69.2%), the UV drained directly to the portal system and those fetuses had neither structural abnormalities nor chromosomal aberrations, while they did not develop any hydrops. In the remaining four cases the DV drained direct to the vena cava (31%) (Figure 2). Three of these cases (23%), were accompanied by increased NT (increased ≥ 99th centile). Two fetuses had aneuploidies (trisomy 21 and trisomy 18), one had a cardiac anomaly, while one developed hydrops and was terminated at 19 weeks. In that case, whole exome sequencing analysis revealed Noonan syndrome.

**Table 1:** Characteristics of the seven fetuses with agenesis of the ductus venosus. CRL=crown-rump-length, NT=nuchal translucency, TOP=termination of pregnancy

	CRL (mm)	NT (mm)	DV draining site	Anomaly	Outcome
Case 1	76.5	2.3	Portal circulation	None	Livebirth
Case 2	56.8	1.4	Portal circulation	None	Livebirth
Case 3	76.7	1.5	Portal circulation	None	Livebirth
Case 4	60.3	1.9	Portal circulation	None	Livebirth
Case 5	67	1.8	Portal circulation	None	Livebirth
Case 6	63.3	1.9	Portal circulation	None	Livebirth
Case 7	69.2	1.7	Portal circulation	None	Livebirth
Case 8	58.2	1.9	Portal circulation	None	Livebirth
Case 9	64	1.6	Portal circulation	None	Livebirth
Case 10	57.8	6.3	Inferior vena cava	Pleural effusions, Trisomy 21	TOP
Case 11	55.9	11.8	Inferior vena cava	Noonan syndrome	TOP
Case 12	61.3	3.9	Inferior vena cava	Multiple Anomalies, Trisomy 18	TOP
Case 13	52.1	2.2	Inferior vena cava	Cardiac anomaly	TOP

## Discussion

As mentioned before, DV plays a crucial role in fetal-maternal normal circulation, since it permits oxygenated maternal blood to bypass the liver and facilitates preferential flow to the left atrium (via the right atrium and foramen ovale). Proper umbilical flow via the DV is essential for the establishment of the intrahepatic portal venous system (IHPVS). Thus, the presence of anatomical variations during fetal development of specific vascular anastomoses between the portal, umbilical, and hepatic-systemic venous systems can lead to altered circulation and even the absence (agenesis) of DV [5].

The prevalence of DV agenesis varies according to the literature and is reported to be between 0.04% and 0.6% [6]. In a screening study of about 66,000 fetuses undergoing routine assessment at 11-13 weeks, the prevalence of DV agenesis was significantly lower (0.04%) compared to our results [4,12]. This is probably due to the fact it is common for many hospitals to refer high-risk fetuses, to tertiary centers for reevaluation and further management. Thus, these results could possibly not represent the true prevalence of DV agenesis since these centers gather many pathological cases [5,13].

Our results indicate a higher prevalence of DV agenesis, specifically one in 650 fetuses (0.15%). In nine out of 13 cases (69%) of DV agenesis, the UV drains into the portal system with a favorable outcome, while in four out of 13 (31%) fetuses with extra-hepatic drainage of the UV, the NT was also increased, and structural and chromosomal anomalies were detected.

The calculated prevalence (0.15%) is higher compared to the literature probably due to the fact that the DV examination - in contrast to previous studies - of all fetuses referred for screening to our unit, was held by two experienced operators, while no cases were needed to be referred to special facilities for further examination. Even though our study had a small volume of cases compared to the studies mentioned above, no other limitations were observed during the study period or with the pregnancies examined (that represented a random population).

In agreement with previous studies, we confirmed that the prognosis of fetuses with DV agenesis depends on the location of the UV drainage, the presence of structural anomalies, and the NT measurement [5-7,13-16]. In our series, the unfavorable outcome was restricted to the fetuses with an extra-hepatic shunt that was all accompanied by increased NT. The fetal phenotype of increased NT and agenesis of the DV with or without cardiac defects has been reported in cases associated with Noonan syndrome as was in one case - patient 11 - of our study [5,17-19].

The results of our study suggest that the prevalence of DV agenesis is higher than previously reported in the routine population undergoing screening at 11-13 weeks. As mentioned before, in the majority of cases (69%), the UV drains to the hepatic circulation and has a benign course with a favorable outcome, making it possible for the ultrasonographer to underestimate DV malformation. In general, agenesis of DV can be underestimated either due to the hemodynamic changes being less significant in the intrahepatic drainage subtype or due to the accompanied fetal anomalies where the ultrasonographer would probably focus.

Various DV agenesis case outcomes have been documented, depending on their correlation with other fetal anomalies and the NT measurement, the PVS development, the kind of umbilical shunt, or the size of the shunt [7,8,19,20]. Rarely documented alone, total portal venous system agenesis is commonly associated with other fetal abnormalities, including heterotaxy, polysplenia, congenital cardiac malformations, Goldenhar syndrome, and chromosomal anomalies. Partial portal venous system agenesis is a benign disorder with a favorable prognosis, as it is also associated with further abnormalities [21]. The absence of DV defines aberrant hemodynamics that may result in the inability of the vitelline veins to transition into the portal system [10]. Portal venous system abnormalities have significant postnatal repercussions with probable adult-onset problems. Given the physiological significance of the two venous systems, the prognosis for agenesis of DV and portal venous system abnormalities varies. We have limited knowledge of the effects of total portal venous system agenesis or partial portal venous system agenesis association in patients with agenesis of DV.

The association of chromosomal abnormalities in cases with DV agenesis is reported in the literature to be between 17.4% and 42.3%. In any suspected case of DV agenesis, a detailed ultrasound scan should be performed to examine fetal anatomy, including fetal echocardiography and determination of the UV drainage. Karyotyping and genetic studies for Noonan syndrome should be offered in particular if NT is increased [10].

## Conclusions

The prevalence of the agenesis of DV is likely to be about 0.15%. The postnatal outcome is unfavorable in the minority of cases with extra-hepatic drainage of the UV, increased NT, and structural defects. In the intrahepatic type, findings may not be distinct. Correct analysis of vessel course is crucial to determine cardiovascular defects and the possibility of poor prognosis. Cardiac failure and hydrops may develop due to overflowing the heart or due to further fetal anomalies; thus, a routine sonographic examination is required in order to detect cardiac failure. Fetal hydrops and the presence of additional congenital anomalies are associated with a worse prognosis, while in isolated cases of DV agenesis, the prognosis is generally good. Genetic studies should be offered with a special focus on Noonan syndrome.
